# Correlation of health-related quality of life for older adults with diabetes mellitus in South Korea: theoretical approach

**DOI:** 10.1186/s12877-023-04186-5

**Published:** 2023-08-14

**Authors:** Gi Won Choi, Sun Ju Chang

**Affiliations:** 1 College of Nursing, Seoul National University, 103 Daehak-ro, Jongno-gu, Seoul, 03080 Republic of Korea; 2 Center for Human-Caring Nurse Leaders for the Future by Brain Korea (BK21) four project, College of Nursing, Seoul National University, 103 Daehak-ro, Jongno-gu, Seoul, 03080 Republic of Korea; 3 College of Nursing and The Research Institute of Nursing Science, Seoul National University, 103 Daehak-ro, Jongno-gu, Seoul, 03080 Republic of Korea

**Keywords:** Older adults, Diabetes mellitus, HIKOD theory, Health-related quality of life.

## Abstract

**Background:**

While some studies have explored the health-related quality of life (HRQOL) of older adults with diabetes mellitus (DM) in South Korea using a theoretical framework, these studies suffer sample-related limitations, as they focus only on a specific subgroup of older adults. To address this gap, this study aimed to investigate the predictors of HRQOL of older adults with DM in South Korea, using extensive national data and based on the theory of Health-Related Quality of Life in South Korean Older Adults with Type 2 Diabetes (The HIKOD theory).

**Methods:**

A secondary data analysis was conducted using data from 1,593 participants aged 65 years and older with DM sourced from the 2015–2019 Korea National Health and Nutrition Examination Survey (KNHANES). The variables included in this study are as follows: demographic factors (gender, age, household income, and education level), disease-specific factors (duration of DM, treatment of DM, and control of HbA1c), barriers (number of comorbidities), resources (living alone status), psychosocial factors (perceived stress), and health-promoting behaviors (physical activity and fundus examination). Considering the complex sampling design employed in this study, statistical analyses including Rao-Scott chi-square tests, correlation analysis, and hierarchical multiple regression analysis were conducted.

**Results:**

Mobility (45.0%) was the HRQOL dimension with which participants experienced the highest number of problems. Number of comorbidities (r = -0.36, *p* < 0.001), living alone status (r_pb_ = 0.16, *p* < 0.001), perceived stress (r_pb_ = 0.14, *p* < 0.001), and physical activity (r_pb_ = 0.12, *p* < 0.001) were correlated with HRQOL. While adjusting for background factors, HRQOL was negatively predicted by higher number of comorbidities (estimate *B* = -0.03, *p* < 0.001), living alone (estimate *B* = -0.03, *p* = 0.043), higher perceived stress (estimate *B* = -0.09, *p* < 0.001), and lower physical activity (estimate *B* = -0.03, *p* < 0.001).

**Conclusion:**

Complex and diverse factors influence HRQOL among older adults with DM in South Korea. To improve their HRQOL, intervention programs that integrally regard HRQOL, along with various predictors, are necessary.

## Background

The meaning of quality of life (QOL) is based on an individual’s perception of their life circumstances within their cultural and value contexts [1]. QOL has been utilized in various contexts, including health status, physical function, symptoms, and psychosocial adaptation. However, the diverse interpretations of QOL presented challenges when comparing research findings or implementing them in practical settings. To address this issue, the concept of health-related quality of life (HRQOL) emerged, narrowing the scope of QOL down specifically to health, disease, and therapeutic aspects [2]. Given its direct association with health, the importance of HRQOL is particularly pronounced among older adults who often experience a decline in functional health and are more vulnerable to various diseases and health issues [3].

According to previous studies, diabetes mellitus (DM) is identified as one of the factors that lower the HRQOL of older adults [4–6]. The prevalence of DM among older populations has been increasing worldwide, and one study projected that the number of individuals aged 65 and above with DM will reach 276.2 million by the year 2045 [7]. In South Korea, DM affects 24.2% of older adults, making it the second most common chronic disease in this population [8]. It is also considered the most burdensome disease in South Korea [9]. For older adults who experience difficulties in acquiring new knowledge and skills due to declining physiological functions, DM poses an additional burden as they need to consistently learn and practice self-management behaviors [10]. Moreover, older adults with DM face a higher risk of microvascular and cardiovascular complications [11], as well as increased risk of mortality [12]. Therefore, the HRQOL of older adults with DM is at risk, and appropriate strategies are required to improve it.

First, a comprehensive understanding of the factors affecting older adults’ HRQOL is necessary [13]. According to previous studies on older adults with DM, their HRQOL was found to be affected by various complex factors. These factors include gender [10, 14, 15], age [14–17], income [14, 17, 18], education level [17], duration of DM [15, 19], treatment of DM [20], HbA1c levels [21], DM-related complications [14, 22], and comorbidities [19, 23]. Additionally, factors such as stress [24, 25], depression [18, 22, 23, 26], self-efficacy [18, 23, 27], social support or family support [26–28], physical activity [14, 25, 29], self-management of DM [10, 15], and functional abilities like instrumental activities of daily living (IADL) or activities of daily living (ADL) [25, 26] were also found to influence HRQOL in older adults with DM.

Surprisingly, only a few studies have applied theoretical frameworks that help to explain the phenomena and relationships between the concepts [30]. Among them, two studies [23, 25] that did employ a theoretical framework were identified: they utilized The Health-Related Quality of Life in South Korean Older Adults with Type 2 Diabetes (HIKOD) theory. The HIKOD theory is a situation-specific theory that was developed based on socio-economic and cultural backgrounds specific to South Korea, aiming to explain the HRQOL of older adults with DM therein [31]. In the first study [23], significant factors influencing HRQOL were found to be DM self-efficacy, depression, DM self-care behaviors, barriers to DM self-care behaviors, and the number of DM-related complications. The second study [25] revealed that IADL, hypertension, arthritis, stress, and physical activity were significant factors affecting HRQOL in older adults with DM. However, it is crucial to note that these two studies had some sample-related limitations. The former study [23] focused on a small number of older adults of each gender, while the latter study [25] focused on older adults with both DM and disabilities. Consequently, the influencing factors on HRQOL were examined in only a subset of South Korean older adults with DM by applying HIKOD theory.

A potential solution to overcome these limitations would be to utilize a large national sample that represents older adults with DM in South Korea more expansively. This way, a more comprehensive understanding of the factors influencing HRQOL in this population could be achieved. Therefore, the current study’s aim was to use data from the Korea National Health and Nutrition Examination Survey (KNHANES) to identify the predictors of the HRQOL of South Korean older adults with DM based on the HIKOD theory.

### Theoretical framework

The HIKOD theory, which was selected as the theoretical framework for this study, suggests that barriers, resources, perceptual factors, psychosocial factors, and health-promoting behaviors influence the HRQOL of South Korean older adults with DM [31]. Specific variables were chosen from the KNHANES dataset based on previous studies and the HIKOD theory.

**Fig. 1:**
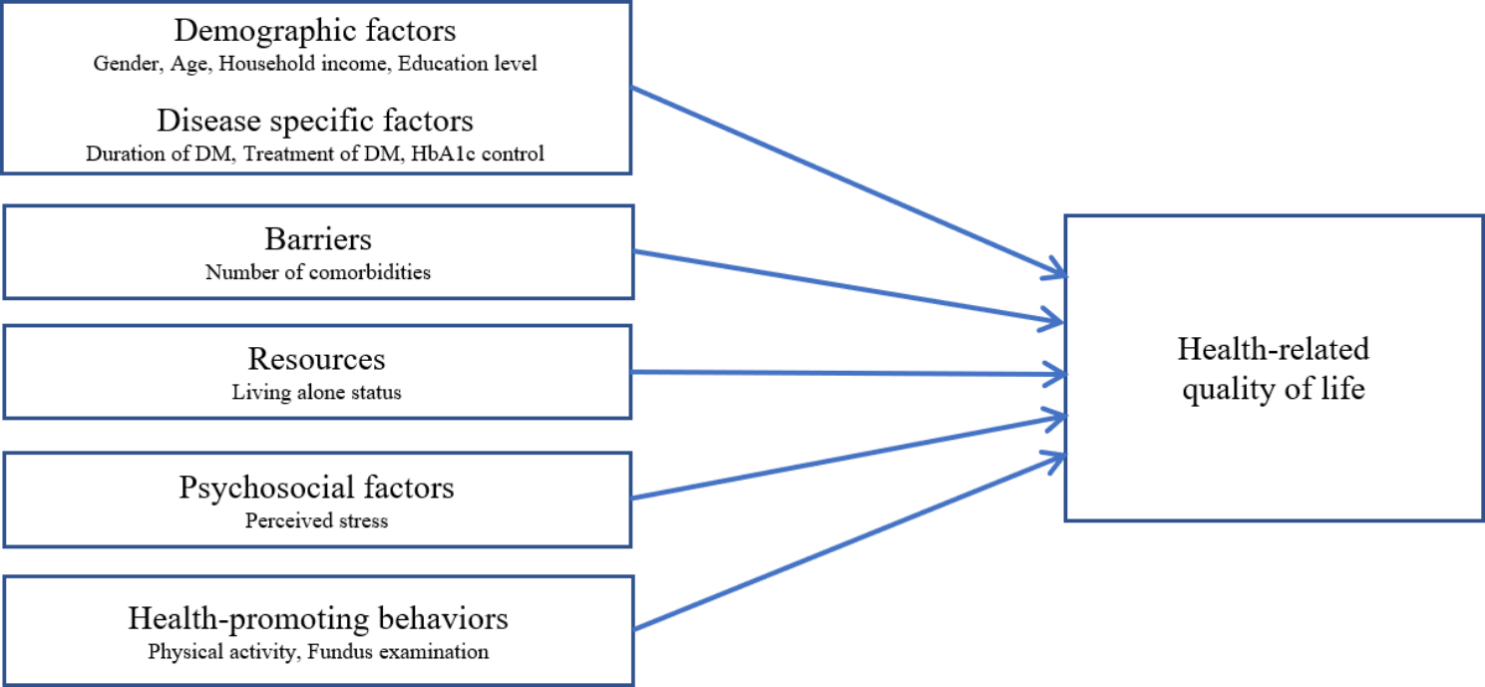
Theoretical framework of the study based on the HIKOD theory [31]. *Note*. DM: Diabetes mellitus.

Firstly, the barrier was considered hindering self-management behaviors related to DM in the HIKOD theory [31]. Here, the number of comorbidities was selected as a relevant factor. Living status (living alone or not) was chosen as a proxy for resources, reflecting family or social support [31]. The perceptual factor related to self-efficacy in DM self-management [31] was excluded due to the absence of corresponding variables in KNHANES. As for the psychosocial factor, the HIKOD theory suggests depression [31], but KNHANES did not specifically measure depression. Therefore, perceived stress was selected, referring to a previous study that applied the HIKOD theory [25]. For health-promoting behaviors corresponding to DM self-management [31], physical activity and fundus examinations were selected. Finally, since the HIKOD theory did not include background factors [31], based on a previous study [23], this study additionally included demographic factors (gender, age, household income, education level) and disease-specific factors (duration of DM, treatment method of DM, HbA1c control). The complete framework, based on these selections, is presented in Fig. 1.

## Methods

### Study design and participants

The present study analyzed data from the KNHANES, a nationwide cross-sectional survey conducted annually by a team of survey experts. The primary objective of KNHANES is to assess the health status, health behavior, and food and nutrition status of South Koreans. To ensure representative sampling, a complex sampling method with a multistage-clustered probability design was employed during the survey [32]. The sampling process involved stratification based on administrative district and housing type, including apartments and general dwellings. Then, 192 primary sampling units (PSUs) per year were selected as clusters. For each PSU, a specific number of households were selected for the survey, with 20 households in 2015, 23 households in 2016–2018, and 25 households from 2019 onwards [33].

**Fig. 2:**
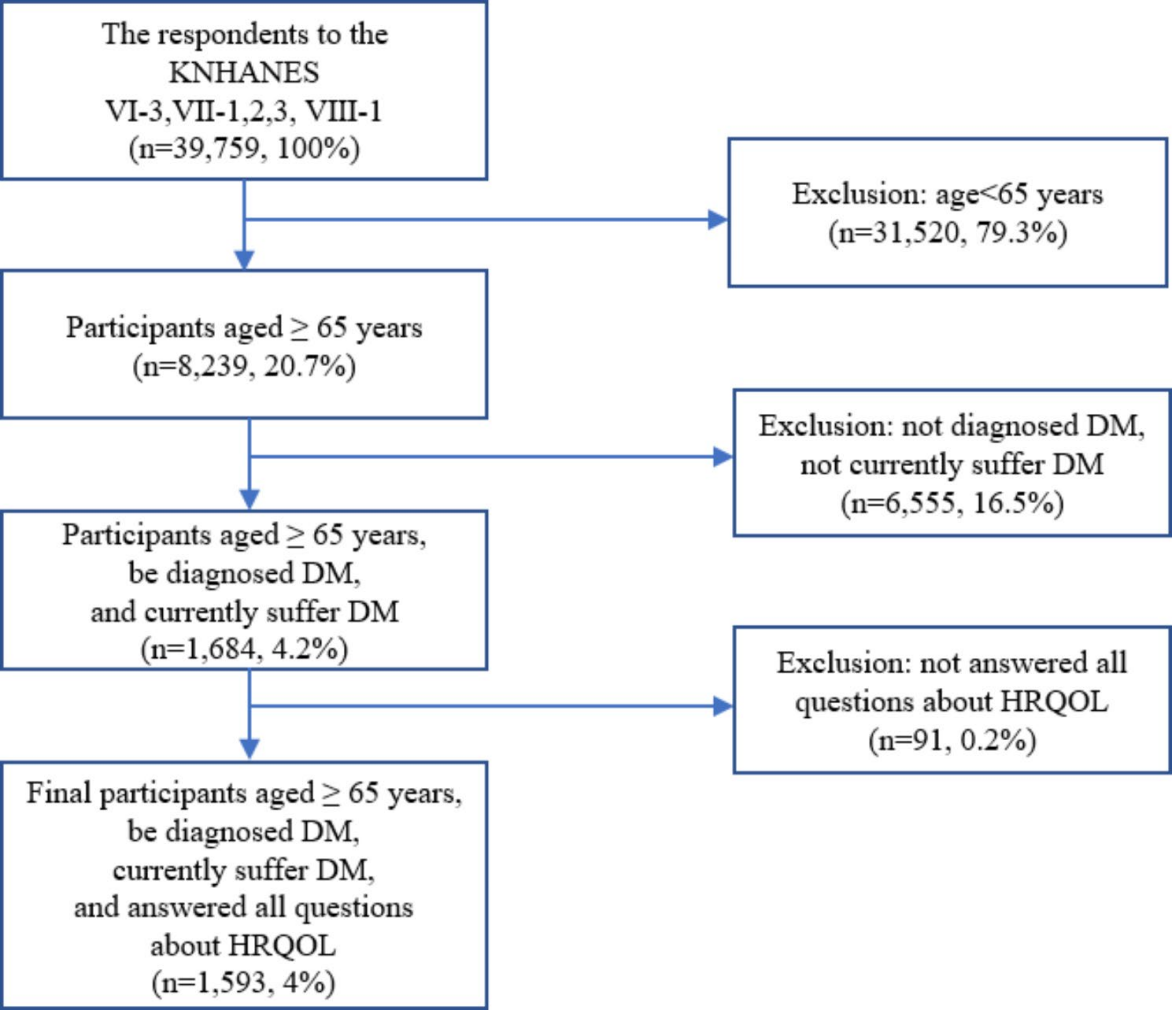
Flowchart for participants. *Note*. DM: Diabetes mellitus; HRQOL: Health-related quality of life; KNHANES: Korea National Health and Nutrition Examination Survey

For this study, data from the 2015 to 2019 period were selected and integrated for analysis. From the initial 2015–2019 KNHANES dataset pool, participants below the age of 65 were initially excluded (79.3%), based on the age criteria defined by the Organization for Economic Cooperation and Development (OECD) for categorizing the older population [34]. Data of participants who were never diagnosed with DM or did not currently suffer from DM were excluded in Step 2 (16.5%). Finally, following the exclusion of those who did not answer all the HRQOL-related questions (0.2%), 1,593 participants (4%) remained as the final sample (Fig. 2).

### Variables

#### Demographic factors

The demographic factors included gender (male and female), age (65–69, 70–74, and 75 years or more), household income, and education level. Household income was categorized into the lowest quartile, 2nd to 3rd quartiles, and the highest quartile based on the household income level adjusted for the number of household members. Education level was classified into four graduation categories: lower/elementary, middle, high, and college/higher education.

#### Disease-specific factors

Duration and treatment of DM and control of HbA1c were measured as diseases-specific factors. The duration of DM referred to the period following the doctor’s diagnosis and was divided into two categories: less than 10 years and 10 years or more. Treatment was classified into three categories: no treatment, only oral hypoglycemic agent (OHA), and other treatments. HbA1c was measured using high-performance liquid chromatography with the Tosoh G8 instrument (Tosoh Corp., Tokyo, Japan) and blood samples. Based on the DM diagnosis criteria outlined by the Korean Diabetes Association, HbA1c levels were classified into two categories: less than 6.5%, and 6.5% or higher [35].

#### Barriers

The number of comorbidities was counted for every chronic disease diagnosed by doctors in the KNHANES: hypertension, dyslipidemia, stroke, myocardial infarction, angina pectoris, osteoarthritis, rheumatoid arthritis, osteoporosis, pulmonary tuberculosis, asthma, thyroid disease, cancer, depression, atopic dermatitis, allergic rhinitis, sinusitis, tympanitis, renal failure, liver cirrhosis, and hepatitis B & C. The number of comorbidities was categorized into three groups: 0–1, 2–3, and more than 4.

#### Resources

Living status was based on the number of household members living together. Participants who answered that they lived alone were considered to be living alone, and other cases were considered as not living alone.

#### Psychosocial factors

Perceived stress was assessed using a single question based on how stress was experienced in daily life. The response options included very high, high, low, and very low. In this study, it was reclassified as high and low.

#### Health-promoting behaviors

The Global Physical Activity Questionnaire (GPAQ) was used to measure physical activity. It was calculated as metabolic equivalents of task-minute per week (MET-min/wk) through moderate or vigorous activities in three domains: work, transport, and recreation. World Health Organization (WHO) recommended that at least 600 MET-min should be achieved; based on this recommended level, participants were divided into two groups: those above (high) and below (low) the recommended level [36]. The fundus examination, one of the eye examinations recommended by the Korean Diabetes Association’s DM guidelines [35], was based on whether participants checked for eye complications caused by DM in the past year.

#### HRQOL

HRQOL was measured using EuroQol-5 Dimensions-3 Level instrument (EQ-5D-3L). There were five dimensions in the EQ-5D-3L: mobility (MO), self-care (SC), usual activities (UA), pain/discomfort (P/D), and anxiety/depression (A/D). Each dimension was rated in three levels: from 1 (“no problem”) to 3 (“extreme problems”). In this study, the EQ-5D index was calculated using the South Korean value set provided by Korea Disease Control and Prevention Agency [37], and the possible range of the index was from − 0.171 to 1.00 [38]. The closer the score to 1, the higher the HRQOL. In the current study, Cronbach’s alpha was calculated to be 0.79.

### Statistical analysis

To consider sampling weights, stratification, and clustering of the KNHANES data, data was analyzed based on a complex sampling design. The weighting of KNHANES considers the extraction rate, response rate, and population distribution, allowing for the estimation of the actual population and calculation of standard errors [39].

Regarding missing data, the rates varied from 0.2 to 7.5% for each variable. To assess the nature of the missing data, a Little’s Missing Completely at Random test was conducted, and it confirmed that the missing values in our data were considered completely random (𝜒^2^ = 1.606, p = 0.658) [40, 41]. Furthermore, following the guidelines provided by KNHANES [39], in cases of item non-response, the missing data were retained as such, but the weight variable for item non-response was included in the analysis to prevent bias in the estimation of standard errors.

According to the analysis results, categorical variables were presented as unweighted frequencies, unweighted percentages, and weighted percentages, while continuous variables were presented as means and standard errors. Rao-Scott chi-square tests were conducted to examine the distribution of perceived problem levels in each dimension of the EQ-5D across each variable. To analyze the correlation between HRQOL and the remaining variables, excluding the participants’ background characteristics (demographic and disease-specific factors), Pearson correlation was used for continuous variables and point-biserial correlation was used for categorical variables. A hierarchical multiple regression analysis using a generalized linear model was conducted to explore the predictors of HRQOL. The analysis was done by incorporating weights, strata, and clusters of the sampling. The nature of population estimation analysis does not require regression analysis assumptions for the sample [39]. However, to further confirm the presence of multicollinearity, which reflects the interrelationships among the independent variables, the variance inflation factor (VIF) was examined. The VIF values, ranging from 1.08 to 3.20, indicated the absence of multicollinearity. The first model included participants’ background factors, including demographic and disease-specific factors. Next, barriers (Second model), resources (Third model), psychosocial factors (Fourth model), and health-promoting behaviors (Fifth model) were added sequentially. For statistical analysis, SPSS version 26.0 software (IBM Corp., Armonk, NY, USA) was used, and statistical significance was defined as a *p*-value < 0.05.

### Ethical consideration

As a secondary data analysis study, this study was exempted from the Institutional Review Board of Seoul National University (no. E2204/001–004). KNHANES was approved by the Korea Disease Control and Prevention Agency research ethics committee (no. 2018-01-03-P-A in 2018, 2018-01-03-C-A in 2019). From 2015 to 2017, since the survey was carried out by the state directly for the benefit of the public, the permission of the research ethics committee was waived [33].

## Results

### Variables’ descriptive statistics

Table 1 presents the descriptive statistics for each variable. After applying the participants’ weights, it was found that 57.8% were women, and the overall mean age was 73.29 ± 0.15 years, with those aged 75 years or older comprising the largest group at 44.9%. About 50.3% fell into the lowest quartile of household income, and 60.9% of participants had education levels below elementary school graduation. The mean duration of DM was 11.66 ± 0.29, and 51.9% had DM for more than 10 years. Among the participants, 80.9% were treated using only OHA; however, 64.0% of participants did not properly control their DM, as their HbA1c levels were 6.5 or higher.

**Table 1:** Descriptive statistics for all variables (unweighted n = 1,593, weighted N = 1,409,433)

Variable	Categories	Unweighted n (%)	Weighted %	Variable	Categories	Unweighted n (%)	Weighted %
**Demographic factors**				**Barrier**			
Gender	Male	704 (44.2)	42.2	Number of	Mean ± SE	2.04 ± 0.04	
	Female	889 (55.8)	57.8	comorbidities	0–1	633 (39.7)	39.8
Age (year)	Mean ± SE	73.29 ± 0.15			2–3	748 (47.0)	46.7
	65–69	442 (27.7)	27.8		≥ 4	212 (13.3)	13.5
	70–74	495 (31.1)	27.3	**Resource**			
	≥ 75	656 (41.2)	44.9	Living alone status	Yes	407 (25.5)	22.9
Household income	Lowest quartile	814 (51.1)	50.3		No	1,186 (74.5)	77.1
	2nd-3rd quartile	648 (40.7)	41.5	**Psychosocial factors**			
	Highest quartile	121 (7.6)	8.1	Perceived stress	Low	1,272 (79.8)	80.7
	Missing	10 (0.6)			High	300 (18.8)	19.3
Education level	≤Elementary	950 (59.6)	60.9		Missing	21 (1.3)	
	Middle	237 (14.9)	14.0	**Health-promoting behaviors**			
	High	279 (17.5)	17.4	Physical activity	Low	1,184 (74.3)	73.6
	≥College	113 (7.1)	7.7		High	409 (25.7)	26.4
	Missing	14 (0.9)		Fundus	Yes	459 (28.8)	28.1
**Disease-specific factors**				examination	No	1,132 (71.1)	71.9
Duration of DM (year)	Mean ± SE	11.66 ± 0.29			Missing	2 (0.1)	
	< 10	766 (48.1)	48.1	**HRQOL**			
	≥ 10	823 (51.7)	51.9	EQ-5D index	Mean ± SE	0.86 ± 0.01	
	Missing	4 (0.3)		Mobility	No problem	875 (54.9)	55.0
Treatment of DM	No treatment	38 (2.4)	2.5		Any problems	718 (45.1)	45.0
	Only OHA	1,300 (81.6)	80.9	Self-care	No problem	1,356 (85.1)	85.3
	Other treatment	255 (16.0)	16.6		Any problems	237 (14.9)	14.7
HbA1c control	< 6.5	538 (33.8)	36.0	Usual activities	No problem	1,192 (74.8)	75.1
	≥ 6.5	935 (58.7)	64.0		Any problems	401 (25.2)	24.9
	Missing	120 (7.5)		Pain/Discomfort	No problem	927 (58.2)	58.9
					Any problems	666 (41.8)	41.1
				Anxiety/Depression	No problem	1,344 (84.4)	85.3
					Any problems	249 (15.6)	14.7

*Note.* DM: Diabetes mellitus; EQ-5D: EuroQol-5 dimensions; HRQOL: Health-related quality of life; OHA: Oral hypoglycemic agent; SE: Standard error

When examining variables based on the HIKOD theory, it was found that the participants had an average of 2.04 ± 0.04 of other chronic diseases, and 46.7% had 2 or 3 diseases in addition to DM. In addition, 22.9% lived alone, and 80.7% reported perceiving a low level of stress. Regarding physical activity, only 26.4% were categorized as having a high level, and 71.9% of participants did not undergo a fundus examination. Finally, the mean EQ-5D index, which represents participants’ HRQOL, was 0.86 ± 0.01. Participants experienced the most problems in the MO dimension (45.0%), followed by P/D (41.1%), UA (24.9%), A/D (14.7%), and SC (14.7%).

### Distribution of perceived problem levels in the dimensions of the EQ-5D

Table 2 presents the distribution of perceived problem levels in each dimension of the EQ-5D across each variable. Compared to men, women were found to experience more problems in all dimensions of the EQ-5D (MO 53.7%, *p* < 0.001; SC 18.1%, *p* < 0.001; UA 29.6%, *p* < 0.001; P/D 48.6%, *p* < 0.001; A/D 16.9%, *p* = 0.006). When considering age groups, older participants had a higher prevalence of problems in MO (56.3%, *p* < 0.001), SC (20.9%, *p* < 0.001), and UA (30.8%, *p* < 0.001) compared to the other age groups. Regarding household income, the lowest quartile exhibited significantly higher percentages of perceived problems in MO (54.2%, *p* < 0.001), SC (17.8%, *p* = 0.009), UA (29.8%, *p* = 0.001), P/D (48.1%, *p* < 0.001), and A/D (18.1%, *p* = 0.001) compared to the other quartile. Concerning education level, except for A/D, participants with an elementary school education level or below reported the highest percentages of problems in MO (53.0%, *p* < 0.001), SC (18.2%, *p* < 0.001), UA (28.9%, *p* < 0.001), and P/D (45.9%, *p* < 0.001). Treatment of DM and HbA1C control did not show significant differences in any dimensions of EQ-5D. However, in relation to the duration of DM, the percentage of problems experienced in the UA was significantly higher in participants with DM for more than 10 years (27.9%, *p* = 0.009) compared to those with DM for less than 10 years. Concerning comorbidities, participants with 4 or more chronic diseases had significantly higher percentages of problems in MO (62.6%. *p* < 0.001), SC (24.8%, *p* < 0.001), UA (43.8%, *p* < 0.001), P/D (66.2%, *p* < 0.001), and A/D (25.3%, *p* < 0.001) compared to those with fewer comorbidities (0–1 or 2–3). Older adults living alone had significantly higher rates of problems in MO (58.6%, *p* < 0.001), SC (19.8%, *p* = 0.007), UA (32.7%, *p* = 0.001), P/D (49.3%, *p* = 0.001), and A/D (21.1%, *p* < 0.001) compared to those living with others. Perceived stress exhibited a significant association with problem rates in all dimensions. Participants in the high stress group reported higher percentages of problems in MO (59.0%, *p* < 0.001), SC (22.1%, *p* < 0.001), UA (39.1%, *p* < 0.001), P/D (58.9%, *p* < 0.001), and A/D (33.0%, *p* < 0.001) compared to those in the low stress group. Similarly, regarding physical activity, older adults with low physical activity levels had significantly higher problem rates in MO (48.5%, *p* < 0.001), SC (16.7%, *p* < 0.001), UA (29.1%, *p* < 0.001), P/D (43.7%, *p* = 0.002), and A/D (15.8%, *p* = 0.046) compared to those with high physical activity levels. Regarding fundus examination, participants who had undergone the examination had a significantly higher problem rate in the P/D (46.9%, *p* = 0.010) compared to those who had not undergone the examination. No statistically significant differences were found in the remaining dimensions.

**Table 2:** Distribution of perceived problem levels in each dimension of the EQ-5D across each variable (unweighted n = 1,593, weighted N = 1,409,433)

Variable		MO		SC		UA		P/D		A/D	
		Any problems (Weighted%)	*p*-value	Any problems (Weighted%)	*p*-value	Any problems (Weighted%)	*p*-value	Any problems (Weighted%)	*p*-value	Any problems (Weighted%)	*p*-value
**Demographic factors**											
Gender	Male	33.1	< 0.001	9.9	< 0.001	18.5	< 0.001	30.8	< 0.001	11.6	0.006
	Female	53.7		18.1		29.6		48.6		16.9	
Age (year)	65–69	30.7	< 0.001	7.2	< 0.001	16.2	< 0.001	36.7	0.058	15.3	0.842
	70–74	41.0		12.1		24.1		40.3		13.8	
	≥ 75	56.3		20.9		30.8		44.3		14.8	
Household income	Lowest quartile	54.2	< 0.001	17.8	0.009	29.8	0.001	48.1	< 0.001	18.1	0.001
	2nd-3rd quartile	38.6		11.6		19.8		35.5		11.9	
	Highest quartile	22.1		10.2		20.1		26.5		8.9	
Education level	≤Elementary	53.0	< 0.001	18.2	< 0.001	28.9	< 0.001	45.9	< 0.001	16.3	0.130
	Middle	31.4		8.5		18.2		36.2		10.8	
	High	32.0		9.3		16.9		31.1		13.7	
	≥College	34.6		9.9		21.2		29.9		11.5	
**Disease-specific factors**											
Duration of DM	< 10	44.5	0.789	12.8	0.113	21.4	0.009	39.7	0.369	14.6	0.951
(year)	≥ 10	45.3		16.3		27.9		42.3		14.7	
Treatment of DM	No treatment	44.2	0.974	15.5	0.985	30.3	0.061	50.6	0.434	19.9	0.190
	Only OHA	44.9		14.6		23.5		40.3		13.8	
	Other treatment	45.7		14.9		31.1		43.5		18.2	
HbA1c control	< 6.5	42.3	0.535	14.3	0.462	24.6	0.599	42.2	0.216	14.8	0.619
	≥ 6.5	44.2		12.8		23.2		38.5		13.8	
**Barrier**											
Number of	0–1	33.3	< 0.001	10.6	< 0.001	17.3	< 0.001	30.0	< 0.001	10.7	< 0.001
comorbidities	2–3	49.9		15.2		25.9		43.3		15.1	
	≥ 4	62.6		24.8		43.8		66.2		25.3	
**Resource**											
Living alone	Yes	58.6	< 0.001	19.8	0.007	32.7	0.001	49.3	0.001	21.1	< 0.001
	No	41.0		13.2		22.6		38.7		12.8	
**Psychosocial factors**											
Perceived stress	Low	41.2	< 0.001	12.3	< 0.001	20.6	< 0.001	36.2	< 0.001	9.9	< 0.001
	High	59.0		22.1		39.1		58.9		33.0	
**Health-promoting behaviors**											
Physical activity	Low	48.5	< 0.001	16.7	< 0.001	29.1	< 0.001	43.7	0.002	15.8	0.046
	High	35.3		9.0		13.2		33.7		11.5	
Fundus	Yes	46.5	0.515	13.8	0.615	26.6	0.423	46.9	0.010	14.5	0.894
examination	No	44.4		15.0		24.3		38.8		14.8	

*Note.* A/D: Anxiety/depression; DM: Diabetes mellitus; MO: Mobility; OHA: Oral hypoglycemic agent; P/D: Pain/discomfort; SC: Self-care; UA: Usual activities

### Correlation with HRQOL

Table 3 displays the results of the correlation analysis between the HRQOL and other variables, excluding demographic and disease-specific factors. The HRQOL demonstrated a significant correlation with the number of comorbidities (r = -0.36, *p* < 0.001), living alone status (r_pb_ = 0.16, *p* < 0.001), perceived stress (r_pb_ = 0.14, *p* < 0.001), and physical activity (r_pb_ = 0.12, *p* < 0.001). The fundus examination had no significant correlation with HRQOL (r_pb_ = 0.00, *p* = 0.835).

**Table 3:** Correlation between variables and HRQOL (unweighted n = 1,593, weighted N = 1,409,433)

	Number of comorbidities		Living alone status		Perceived stress		Physical activity		Fundus examination	
	r	*p*-value	r_pb_	*p*-value	r_pb_	*p*-value	r_pb_	*p*-value	r_pb_	*p*-value
**HRQOL**	-0.36	< 0.001	0.16	< 0.001	0.14	< 0.001	0.12	< 0.001	0.00	0.835

*Note.* HRQOL: Health-related quality of life

### Predictors for HRQOL

Table 4 demonstrates the results obtained following a hierarchical multiple regression analysis to find related predictors in older adults with DM in South Korea. Among the variables, fundus examination was excluded from regression as it was not significantly correlated with HRQOL in the correlation analysis. In Model 1, males (estimate *B* = 0.05, *p* < 0.001), highest quartile in household income (estimate *B* = 0.05, *p* = 0.003), middle school education levels (estimate *B* = 0.04, *p* = 0.009), high school education levels (estimate *B* = 0.03, *p* = 0.005), and college or higher education levels (estimate *B* = 0.04, *p =* 0.004) were found to be significant positive factors of HRQOL in older adults with DM, while age (estimate *B* = -0.01, *p* < 0.001) negatively affected HRQOL. The explanatory power of the model was 9.7% (*p* < 0.001). In Model 2, when adjusting the demographic and disease-specific factors, an increased number of comorbidities (estimate *B* = -0.03, *p* < 0.001) was identified as significantly associated with poor HRQOL, and the explanatory power was increased to 14.3% (*p* < 0.001). Living alone (estimate *B* = -0.03, *p* = 0.043) and high perceived stress (estimate *B* = -0.09, *p* < 0.001) predicted the HRQOL negatively, which accounted for additional 0.4% (*p =* 0.004) in Model 3, and 3.3% (*p* < 0.001) in Model 4 respectively, while controlling the previous models’ factors. Finally, in Model 5, following adjustment for other variables, low physical activity (estimate *B* = -0.03, *p* < 0.001) was included as a predictor for poor HRQOL. The explanatory power of this model was significantly increased by 0.7% (*p* < 0.001) compared to the prior model, with the final explanatory power reaching 18.7% (*p* < 0.001).

**Table 4:** Hierarchical Multiple Regression Analysis of HRQOL (unweighted n = 1,593, weighted N = 1,409,433)

Variable		Model 1		Model 2		Model 3		Model 4		Model 5	
		Estimate *B*	*p*-value	Estimate *B*	*p*-value	Estimate *B*	*p*-value	Estimate *B*	*p*-value	Estimate *B*	*p*-value
Constant		1.16	< 0.001	1.18	< 0.001	1.19	< 0.001	1.27	< 0.001	1.28	< 0.001
Gender (ref: female)	Male	0.05	< 0.001	0.03	0.005	0.02	0.016	0.02	0.052	0.02	0.058
Age (year)		-0.01	< 0.001	0.00	< 0.001	0.00	< 0.001	-0.01	< 0.001	0.00	< 0.001
Household income (ref: lowest quartile)	Highest quartile	0.05	0.003	0.04	< 0.004	0.04	0.022	0.03	0.041	0.03	0.041
	2nd-3rd quartile	0.02	0.085	0.02	0.107	0.01	0.357	0.01	0.656	0.00	0.775
Education level (ref: ≤elementary)	Middle	0.04	0.009	0.03	0.013	0.03	0.013	0.03	0.029	0.03	0.050
	High	0.03	0.005	0.03	0.013	0.03	0.013	0.02	0.021	0.02	0.032
	≥College	0.04	0.004	0.04	0.004	0.04	0.003	0.04	0.008	0.03	0.021
Duration of DM (year)		0.00	0.316	0.00	0.645	0.00	0.709	0.00	0.956	0.00	0.881
Treatment of DM (ref: no treatment)	Only OHA	0.04	0.176	0.05	0.031	0.05	0.031	0.03	0.214	0.02	0.239
	Other treatment	0.02	0.515	0.04	0.147	0.04	0.159	0.01	0.615	0.01	0.706
HbA1c control (ref: <6.5)	≥ 6.5	0.01	0.578	0.00	0.888	0.00	0.843	-0.01	0.580	-0.01	0.556
Number of comorbidities				-0.03	< 0.001	-0.03	< 0.001	-0.02	< 0.001	-0.02	< 0.001
Living alone status (ref: no)	Yes					-0.03	0.043	-0.03	0.039	-0.03	0.048
Perceived stress (ref: low)	High							-0.09	< 0.001	-0.09	< 0.001
Physical activity (ref: high)	Low									-0.03	< 0.001
Wald F (*p*-value)		12.47(< 0.001)		15.40(< 0.001)		14.53(< 0.001)		15.35(< 0.001)		15.08(< 0.001)	
R^2^		0.097		0.143		0.147		0.180		0.187	
R^2^ change (*p*-value)				0.046(< 0.001)		0.004(0.004)		0.033(< 0.001)		0.007(< 0.001)	

*Note.* DM: Diabetes mellitus; OHA: Oral hypoglycemic agent

## Discussion

The present study was conducted to identify the predictors of HRQOL among older adults with DM in South Korea, using 2015–2019 KNHANES data and HIKOD theory. After adjusting for background factors, a regression analysis revealed that the number of comorbidities, living alone status, perceived stress, and physical activity were predictors of HRQOL South Korean older adults with DM.

MO was identified as the dimension of HRQOL in which participants experienced the most difficulties, which was similar to a study in Vietnam where MO also ranked second [15]. However, a study conducted in Bangladesh with adult patients reported a lower ranking for MO [42]. This difference may be attributed to the challenges faced by older people in terms of mobility, such as reduced muscle strength, decreased flexibility, and limited exercise capacity associated with the aging process [43]. In our study, participants aged 75 years or older encountered more problems with MO compared to other age groups, suggesting that the impact of aging becomes more pronounced with advancing age. This negatively influenced overall HRQOL, as evidenced by Model 1 of the regression analysis in our study. Therefore, to comprehensively assess the HRQOL of older adults with DM in South Korea, it is crucial to gain a comprehensive understanding of the aging characteristics specific to this population.

In model 1—which included background factors—gender, household income, and education level were also found to be significant variables affecting the HRQOL of older adults with DM in South Korea. Consistent with previous studies [10, 15], men in this study exhibited higher HRQOL compared to women. This difference could be attributed to cultural factors, as older adult men in South Korea often receive support from their spouses, including assistance with meal preparation [10]. On the other hand, older adult women tend to prioritize family-oriented responsibilities such as meal preparation and caring for grandchildren, potentially neglecting their own health, which may contribute to lower HRQOL [23]. Additionally, the longer life expectancy of women than men [8] may also play a role, considering that this study found age to have a negative impact on HRQOL. Therefore, it is crucial to develop strategies specifically targeted at improving the HRQOL of older adult women with DM in South Korea.

In this study, participants with the highest household income had higher levels of HRQOL compared to those in the lowest income quartile, which aligns with previous research findings [14, 17, 18]. This association may be attributed to the lower financial burden of managing DM, as higher household income presents fewer challenges in affording necessary treatments [44]. Moreover, South Korea has the highest older population poverty rate of 43.4% among OECD countries [45], and in this study, more than half of the participants belonged to the lowest income level, highlighting a serious social problem. To address this issue, various economic support measures such as job provisions and treatment cost subsidies at the national level are needed. By implementing such measures, it is expected that HRQOL can be improved for older adults with DM.

This study demonstrated that participants with above-elementary-school education levels had higher HRQOL, which was consistent with other studies [46, 47]. Additionally, participants who were elementary school graduates or lower experienced approximately double as much SC problems as the other groups. A previous study explained that a person’s comprehension of diseases increased with education level, which improved treatment awareness and self-management skills [46]. Therefore, to improve their HRQOL, older adults with DM who have lower education levels should be provided more intensive education on SC.

According to the HIKOD theory component, the first negative predictor of HRQOL in older adults with DM in South Korea was the number of comorbidities. This finding was similar to that of a previous research indicating that the presence of comorbidities has a detrimental impact on HRQOL [48]. In particular, the majority of the participants were already receiving DM medication in the form of OHA in our study. Consequently, those with comorbidities had to take additional medications as part of their treatment, which could impose a burden on them [11]. Moreover, the use of multiple medications may lead to potential health risks due to side effects or drug interactions, ultimately contributing to a decline in HRQOL [48]. Thus, for older adults with DM suffering from comorbidities, it is essential to acknowledge drug use as a burden and closely monitor them to see if any additional issues arise or their HRQOL deteriorates.

Living alone status was another negative predictor of participants’ HRQOL. It could be a critical predictor for older adults with DM rather than for those without, as they had to take care of themselves as well as self-manage their DM. In contrast, the emotional support that patients with DM receive from family or people around them positively affects their self-management [27], and enhanced self-management positively affects their HRQOL [10]. For older adults living alone, the level of emotional support could be lower compared to that of older adults living together as they lack families, which is considered the closest social unit in South Korean society. To improve the HRQOL for older adults with DM living alone, efforts should be made to increase their level of emotional support through a community approach.

Another negative predictor of HRQOL in South Korean older adults with DM was perceived stress: highly stressed participants had poorer HRQOL, which was consistent with previous studies [24, 25]. The inclusion of stress in Model 4 resulted in the previously significant gender variable becoming insignificant, suggesting that the strong impact of stress superseded the significance of gender in this framework. Stress has been found to have a detrimental effect on the emotional well-being and HRQOL of older adults [49]. In South Korea, where traditional family systems and Confucian ideals shape societal norms, older adults face various stresses related to aging, retirement, and changing roles within the household [49]. Furthermore, managing DM itself, which requires significant lifestyle changes including dietary modifications, can act as a major stressor for older adults [50]. Although the proportion of participants with high stress in this study was approximately one-fifth, the vulnerabilities of older adults with DM in South Korea to stress should not be overlooked. Therefore, recognizing situations that make them susceptible to stress and developing diverse strategies to help them effectively cope with stress are crucial steps in enhancing their HRQOL.

Physical activity was also identified as a significant influencing factor of HRQOL. Participants with low physical activity had lower HRQOL compared to those with high activity, which was consistent with previous studies [14, 29]. Exercise, a part of the physical activities recommended for older adults with DM, is helpful in strengthening muscles, functional ability, and cardiopulmonary function, depending on the type [51]. In addition, physical activity is an important aspect for older adults with DM rather than those without; increasing physical activity is effective in reducing, and thus controlling, blood glucose [47]. In this study, the percentage of low physical activity was high in the group that experienced problems in all dimensions of HRQOL. Considering a previous study that stated that physical activity helped both physical and mental health and increased HRQOL [47], education is needed to develop competency so that older adults with DM themselves can plan appropriate physical activities according to their physical situations.

Surprisingly, although the present study focused on older adults with DM, disease-specific factors were not shown to be significant factors of HRQOL in the final model. Additionally, there was no correlation between fundus examination and HRQOL. Even within the HRQOL dimensions, significant differences were only observed in the UA for the duration of the DM and P/D for the fundus examination. This could potentially be attributed to the measurement tool used. In this study, the HRQOL was measured using non-specific tools, such as EQ-5D; thus, it may have not captured the characteristics of DM sensitively [52]. Considering that the results of existing studies were different [15, 52], repeated studies are required to determine whether DM-related characteristics affected HRQOL in older adults with DM.

There are certain limitations associated with the present study. First, as KNHANES assessed the overall health of South Koreans [32], there was a lack of information on the detailed factors for DM. For example, DM classification (i.e., Type 1 or 2) or hypoglycemic experience were not captured in KNHANES. Future studies should include more diverse DM-specific variables. Second, in a similar context, HRQOL was measured using the EQ-5D-3LL, a non-specific tool, rather than a DM-specific tool. Therefore, the effect of DM was not properly reflected in the HRQOL [52]. Third, most of the variables were measured as a single question, so there were limitations in additional statistical analysis. It is necessary to analyze the same using measured variables based on multiple questions that have secured validity and reliability [53]. Fourth, the present study combined each year’s KNHANES from 2015 to 2019; some variables could not be analyzed as the subject of measurement was different depending on the year even for the same variable. For example, data from the comprehensive eye examination, that could be included together with the fundus examination in the health-promoting behaviors, were collected from subjects under the age of 65 from 2015 to 2016. Finally, since KNHANES is a cross-sectional data, there was a limit to the interpretation of the causal relationship between the variables [54].

Despite the limitations, the present study possesses a significant strength as it addresses the previous studies’ limitations by utilizing large national data to identify the factors influencing the HRQOL among older adults with DM in South Korea. Furthermore, this study offers the advantage of systematically examining predictive factors based on the theoretical framework of the HIKOD theory. The findings of this study hold valuable implications for the development of intervention programs aimed at improving the HRQOL of older adults with DM in South Korea and can also be applied to countries facing similar contexts.

## Conclusions

This study employed national statistics to investigate the predictors of HRQOL among older adults with DM in South Korea, using the HIKOD theory as a framework. Among the HRQOL dimensions, MO was identified as the most problematic dimension for this population. Perceived problem levels in all HRQOL dimensions differed according to gender, household income, number of comorbidities, living alone status, perceived stress, and physical activity. Correlations were observed between HRQOL and the number of comorbidities, living alone status, perceived stress, and physical activity. After adjusting for background factors, higher number of comorbidities, living alone status, higher perceived stress, and lower physical activity emerged as negative predictors of HRQOL in older adults with DM in South Korea. Based on these findings, it is recommended to conduct further diverse research on the HRQOL of older adults with DM in South Korea, utilizing more specific and objective variables and tools that focus specifically on DM-related factors. Additionally, the development of intervention programs that comprehensively consider the various predictors identified in this study is necessary to effectively promote and improve the HRQOL of older adults with DM in South Korea.

## Data Availability

The KNHANES data used in this study can be used for research purposes by accessing the KNHANES website: https://knhanes.kdca.go.kr/knhanes/eng/index.do.

## Abbreviations

ADL: Activities of daily living
A/D: Anxiety/depression
DM: Diabetes mellitus
EQ-5D: EuroQol-5 Dimensions
GPAQ: Global Physical Activity Questionnaire
HIKOD: Health-Related Quality of Life in South Korean Older Adults with Type 2 Diabetes
HRQOL: Health-related quality of life
IADL: Instrumental activities of daily living
KNHANES: Korea National Health and Nutrition Examination Survey
MO: Mobility
OHA: Oral hypoglycemic agent
P/D: Pain/discomfort
PSU: Primary sampling unit
QOL: Quality of life
SC: Self-care
UA: Usual activities
VIF: Variance inflation factor
WHO: World Health Organization

## References

[1] World Health Organization Assessment Group (1996). What quality of life?. World Health Forum.

[2] Ferrans CE, Zerwic JJ, Wilbur JE, Larson JL. Conceptual model of health-related quality of life. J Nurs Scholarsh 2005:37(4):336–42. 10.1111/j.1547-5069.2005.00058.x. 10.1111/j.1547-5069.2005.00058.x 16396406

[3] Etxeberria I, Urdaneta E, Galdona N (2019). Factors associated with health-related quality of life (HRQoL): differential patterns depending on age. Qual Life Res.

[4] Hajian-Tilaki K, Heidari B, Hajian-Tilaki A (2016). Solitary and combined negative influences of diabetes, obesity and hypertension on health-related quality of life of elderly individuals: a population-based cross-sectional study. Diabetes Metab Syndr.

[5] Zhuang Y, Ma QH, Pan CW, Lu J. Health-related quality of life in older chinese patients with diabetes. PLoS One 2020:15(2):e0229652. 10.1371/journal.pone.0229652. 10.1371/journal.pone.0229652 PMC7046237 32106232

[6] Tian S, Wang R, Qian M, Liu L, Shao Z, Wu C, Sun J (2021). The association between diabetes mellitus and HRQoL of older people in Shanghai. BMC Geriatr.

[7] Sinclair A, Saeedi P, Kaundal A, Karuranga S, Malanda B, Williams R. Diabetes and global ageing among 65–99-year-old adults: findings from the International Diabetes Federation Diabetes Atlas. Diabetes Res Clin Pract. 2020;162108078. 10.1016/j.diabres.2020.108078. 10.1016/j.diabres.2020.108078 32068097

[8] Ministry of Health and Welfare. 2020 National Survey of Living Conditions and Welfare Needs of Korean Older Persons. 2020. http://www.mohw.go.kr/react/jb/sjb030301vw.jsp?PAR_MENU_ID=03&MENU_ID=032901&CONT_SEQ=366496. Accessed 9 April 2022.

[9] Jung Y-S, Kim Y-E, Park H, Oh I-H, Jo M-W, Ock M, Go D-S, Yoon S-J (2021). Measuring the burden of disease in Korea, 2008–2018. J Prev Med Public Health.

[10] Kim HS, Kim KS. Health-Related Quality-of-life and diabetes self-care activity in Elderly patients with diabetes in Korea. J Community Health 2017:42(5):998–1007. 10.1007/s10900-017-0347-2. 10.1007/s10900-017-0347-2 28432547

[11] Kirkman MS, Briscoe VJ, Clark N, Florez H, Haas LB, Halter JB, Huang ES, Korytkowski MT, Munshi MN, Odegard PS. Diabetes in older adults. Diabetes Care 2012:35(12):2650–64. 10.2337/dc12-1801. 10.2337/dc12-1801 PMC3507610 23100048

[12] Huang ES, Laiteerapong N, Liu JY, John PM, Moffet HH, Karter AJ. Rates of complications and mortality in older patients with diabetes mellitus: the diabetes and aging study. JAMA Intern Med. 2014:174(2):251–8. 10.1001/jamainternmed.2013.12956. 10.1001/jamainternmed.2013.12956 PMC3950338 24322595

[13] Dickerson F, Wohlheiter K, Medoff D, Fang L, Kreyenbuhl J, Goldberg R, Brown C, Dixon L. Predictors of quality of life in type 2 diabetes patients with schizophrenia, major mood disorder, and without mental illness. Qual Life Res. 2011:20:1419–25. 10.1007/s11136-011-9888-5. 10.1007/s11136-011-9888-5 21424900

[14] Tang WL, Wang YM, Du WM, Cheng NN, Chen BY. Assessment of quality of life and relevant factors in elderly diabetic patients in the Shanghai community. Pharmacoepidemiol Drug Saf 2006:15(2):123–30. 10.1002/pds.1166. 10.1002/pds.1166 16294368

[15] Nguyen HTT, Moir MP, Nguyen TX, Vu AP, Luong LH, Nguyen TN, Nguyen LH, Tran BX, Tran TT, Latkin CA (2018). Health-related quality of life in elderly diabetic outpatients in Vietnam. Patient Prefer Adherence.

[16] Kang Y, Park K (2020). Health-related quality of life in elderly patients with diabetes mellitus according to age: based on Korea National Health and Nutrition Examination Survey. J Nutr Health.

[17] Shamshirgaran SM, Stephens C, Alpass F, Aminisani N. Longitudinal assessment of the health-related quality of life among older people with diabetes: results of a nationwide study in New Zealand. BMC Endocr Disord 2020:20:1–9. 10.1186/s12902-020-0519-4. 10.1186/s12902-020-0519-4 PMC7059720 32138698

[18] Sari Y, Isworo A, Upoyo AS, Taufik A, Setiyani R, Swasti KG, Haryanto H, Yusuf S, Nasruddin N, Kamaluddin R (2021). The differences in health-related quality of life between younger and older adults and its associated factors in patients with type 2 diabetes mellitus in Indonesia. Health Qual Life Outcomes.

[19] Liu T, Man X, Miao X. Geriatric syndromes and the cumulative impacts on quality of life in older people with type 2 diabetes mellitus. Int J Diabetes Dev Ctries. 2021:41:148–55. 10.1007/s13410-020-00848-x.

[20] Brown DW, Balluz LS, Giles WH, Beckles GL, Moriarty DG, Ford ES, Mokdad AH (2004). Diabetes mellitus and health-related quality of life among older adults: findings from the behavioral risk factor surveillance system (BRFSS). Diabetes Res Clin Pract.

[21] Aro A-K, Karjalainen M, Tiihonen M, Kautiainen H, Saltevo J, Haanpää M, Mäntyselkä P. Glycemic control and health-related quality of life among older home-dwelling primary care patients with diabetes. Prim Care Diabetes. 2017:11(6):577–82. 10.1016/j.pcd.2017.07.001. 10.1016/j.pcd.2017.07.001 28754430

[22] Maatouk I, Wild B, Wesche D, Herzog W, Raum E, Müller H, Rothenbacher D, Stegmaier C, Schellberg D, Brenner H (2012). Temporal predictors of health-related quality of life in elderly people with diabetes: results of a german cohort study. PLoS ONE.

[23] Choi JS, Kim BH, Chang SJ (2015). Gender-specific factors influencing diabetes self-care behaviors and health-related quality of life among older adults with type 2 diabetes in South Korea. Res Gerontol Nurs.

[24] Shin JW, Park YK, Suh SR, Kim JE. Factors influencing quality of life in elderly diabetic patients of Korea: analysis from the Korea National Health and Nutrition Examination Survey in 2008. J Korea Gerontological Soc. 2011:31(3):479–81.

[25] Jeong HN, Chang SJ (2021). Health-Related Quality of Life among Elderly individuals with both diabetes and disabilities in Korea: results from a nationally Representative Survey. Int J Gerontol.

[26] Johari N, Manaf ZA, Ibrahim N, Shahar S, Mustafa N. Predictors of quality of life among hospitalized geriatric patients with diabetes mellitus upon discharge. Clin Interv Aging 2016:11:1455–61. 10.2147/CIA.S105652. 10.2147/CIA.S105652 PMC5074738 27799751

[27] Kim YJ, Seo NS, Kim SJ, Park IS, Kang SJ. Quality of life and its correlated factors among elderly people with diabetes in a community. Korean J Health Service Manage. 2014:8(1):75–86. 10.12811/kshsm.2014.8.1.075.

[28] Choi Y, Kim H, Kim M, Shim M, Lee J, Kim M, Jung C (2002). Family support and life quality in elderly diabetic patients. J Korean Diabetes.

[29] Huang CC, Hsu CC, Chiu CC, Lin HJ, Wang JJ, Weng SF (2020). Association between exercise and health-related quality of life and medical resource use in elderly people with diabetes: a cross-sectional population-based study. BMC Geriatr.

[30] Polit DF, Beck CT (2021). Theoretical frameworks. Nursing research: Generating and assessing evidence for nursing practice.

[31] Chang SJ, Im EO (2014). Development of a situation-specific theory for explaining health-related quality of life among older south korean adults with type 2 diabetes. Res Theory Nurs Pract.

[32] Korea Disease Control and Prevention Agency. Korea National Health & Nutrition Examination Survey. n.d. https://knhanes.kdca.go.kr/knhanes/eng/index.do. Accessed 10 Sep 2022.

[33] Korea Disease Control and Prevention Agency. Raw Data User Guide. 2023. https://knhanes.kdca.go.kr/knhanes/sub03/sub03_06_02.do. Accessed 10 July 2023.

[34] Organization for Economic Cooperation and Development. Elderly population. n.d. https://data.oecd.org/pop/elderly-population.htm. Accessed 21 May 2023.

[35] Korean Diabetes Association. Clinical Practice Guidelines for Diabetes. 2021. https://www.diabetes.or.kr/bbs/?code=guide&mode=view&number=853&page=1&code=guide. Accessed 16 February 2023.

[36] World Health Organization. Global physical activity questionnaire (GPAQ) analysis guide. 2012. https://www.who.int/docs/default-source/ncds/ncd-surveillance/gpaq-analysis-guide.pdf. Accessed 15 April 2022.

[37] Lee YK, Nam HS, Chuang LH, Kim KY, Yang HK, Kwon IS, Kind P, Kweon SS, Kim YT (2009). South korean time trade-off values for EQ-5D health states: modeling with observed values for 101 health states. Value Health.

[38] Hong JY, Kim SY, Chung KS, Kim EY, Jung JY, Park MS, Kang YA, Kim SK, Chang J, Kim YS (2015). Factors associated with the quality of life of korean COPD patients as measured by the EQ-5D. Qual Life Res.

[39] Korea Disease Control and Prevention Agency. Analysis Guidelines (SPSS). 2013. https://knhanes.kdca.go.kr/knhanes/sub03/sub03_06_02.do. Accessed 10 September 2022.

[40] Little RJ (1988). A test of missing completely at random for multivariate data with missing values. J Am Stat Assoc.

[41] Mirzaei A, Carter SR, Patanwala AE, Schneider CR (2022). Missing data in surveys: key concepts, approaches, and applications. Res Social Adm Pharm.

[42] Saleh F, Ara F, Mumu SJ, Hafez MA. Assessment of health-related quality of life of bangladeshi patients with type 2 diabetes using the EQ-5D: a cross-sectional study. BMC Res Notes. 2015:8(1):1–8. 10.1186/s13104-015-1453-9. 10.1186/s13104-015-1453-9 PMC4588249 26420245

[43] Prasad L, Fredrick J, Aruna R. The relationship between physical performance and quality of life and the level of physical activity among the elderly. J Educ Health Promot. 2021:10:68. 10.4103/jehp.jehp_421_20. 10.4103/jehp.jehp_421_20 PMC8057187 34084815

[44] Natarajan J, Mokoboto-Zwane S (2022). Health-related quality of life and domain-specific associated factors among patients with Type2 diabetes mellitus in south India. Rev Diabet Stud.

[45] Organization for Economic Cooperation and Development. Old-age income poverty. 2021. https://www.oecd-ilibrary.org/sites/d76e4fad-en/index.html?itemId=/content/component/d76e4fad-en. Accessed 31 May 2023.

[46] Javanbakht M, Abolhasani F, Mashayekhi A, Baradaran HR, Jahangiri noudeh Y. Health related quality of life in patients with type 2 diabetes mellitus in Iran: a national survey. PLoS One 2012:7(8):e44526. 10.1371/journal.pone.0044526. 10.1371/journal.pone.0044526 PMC3431351 22952989

[47] Shetty A, Afroz A, Ali L, Siddiquea BN, Sumanta M, Billah B. Health-related quality of life among people with type 2 diabetes mellitus–A multicentre study in Bangladesh. Diabetes Metab Syndr 2021:15(5):102255. 10.1016/j.dsx.2021.102255. 10.1016/j.dsx.2021.102255 34479101

[48] Gebremedhin T, Workicho A, Angaw DA (2019). Health-related quality of life and its associated factors among adult patients with type II diabetes attending Mizan Tepi University Teaching Hospital, Southwest Ethiopia. BMJ Open Diabetes Res Care.

[49] Bae J, Kim H, Yang M, Kim H, Kim J, Lim H (2013). Stress and management strategies in korean elderly. Crisisonomy.

[50] Falco G, Pirro PS, Castellano E, Anfossi M, Borretta G, Gianotti L (2015). The relationship between stress and diabetes mellitus. J Neurol Psychol.

[51] Cadore EL, Izquierdo M (2015). Exercise interventions in polypathological aging patients that coexist with diabetes mellitus: improving functional status and quality of life. Age.

[52] Nezu S, Okamoto N, Morikawa M, Saeki K, Obayashi K, Tomioka K, Komatsu M, Iwamoto J, Kurumatani N (2014). Health-related quality of life (HRQOL) decreases independently of chronic conditions and geriatric syndromes in older adults with diabetes: the Fujiwara-kyo Study. J Epidemiol.

[53] Park EO, Choi SJ (2013). Prevalence of suicidal ideation and related risk factors among korean adults. J Korean Acad Psych Mental Health Nurs.

[54] Polit DF, Beck CT (2021). Planning a nursing study. Nursing research: Generating and assessing evidence for nursing practice.

